# *Mycoplasma pneumoniae*‐Associated Mucositis: A Diagnostic Dilemma

**DOI:** 10.1002/ccr3.71338

**Published:** 2025-10-22

**Authors:** Mahesh Mathur, Sumit Paudel, Nabita Bhattarai, Sambidha Karki, Shilpa Maharjan, Sandhya Regmi

**Affiliations:** ^1^ Department of Dermatology College of Medical Sciences Teaching Hospital Bharatpur Nepal

**Keywords:** Fuchs syndrome, general dermatology, *Mycoplasma pneumonia*, *Mycoplasma pneumoniae* ‐associated mucositis

## Abstract

Fuchs syndrome is a mucosal variant of Stevens‐Johnson syndrome (SJS) without cutaneous lesions, mostly affecting the mucosa of the mouth, conjunctiva, and genitalia, that occurs in a background of *Mycoplasma pneumoniae* and herpes simplex infection. Treatment with antibiotics such as macrolides, tetracycline, or fluoroquinolones has been shown to limit the pulmonary disease, but it is unclear whether the incidence or severity of the mucocutaneous eruption is reduced. In the absence of cutaneous manifestations, the patient often seeks multiple specialists, including ophthalmologists, dentists, otolaryngologists, gynecologists, or urologists, so a suspicion of *Mycoplasma* in such cases can avoid delay in diagnosis and adverse sequelae. We hereby report a case of *Mycoplasma pneumoniae* ‐associated Fuchs syndrome which showed significant improvement with oral antibiotics.

**Summary**

-
*Mycoplasma pneumoniae* ‐associated mucositis is a rare variant of Stevens‐Johnson syndrome (SJS) characterized by two or more mucosal involvements without skin lesions.
- In the absence of cutaneous manifestations, the patient often seeks multiple specialists; a suspicion of *Mycoplasma* in such cases can avoid delay in diagnosis and adverse sequelae.

**Abbreviations**
HSV: herpes simplex virus
MIRM: *Mycoplasma*‐induced rash and mucositis
MPAM:
*Mycoplasma pneumoniae* ‐associated mucositis
PCR: polymerase chain reaction
RIME: reactive infectious mucocutaneous eruption
RIRM: respiratory infection‐induced rash and mucositis
SJS: Stevens‐Johnson syndrome

## 1 Introduction

Fuchs syndrome is a mucosal variant of Stevens‐Johnson syndrome (SJS) without cutaneous lesions, mostly affecting the mucosa of the mouth, conjunctiva, and genitalia [1]. The disease is more common in children and occurs in a background of *Mycoplasma pneumoniae* and herpes simplex infection. This entity has been variably described as incomplete SJS, atypical SJS, or erythema multiforme (EM) major without skin lesions [1, 2]. We hereby report a case of *Mycoplasma pneumoniae* ‐associated Fuchs syndrome, which showed drastic improvement with oral antibiotics.

## 2 Case Presentation

**FIGURE 1:**
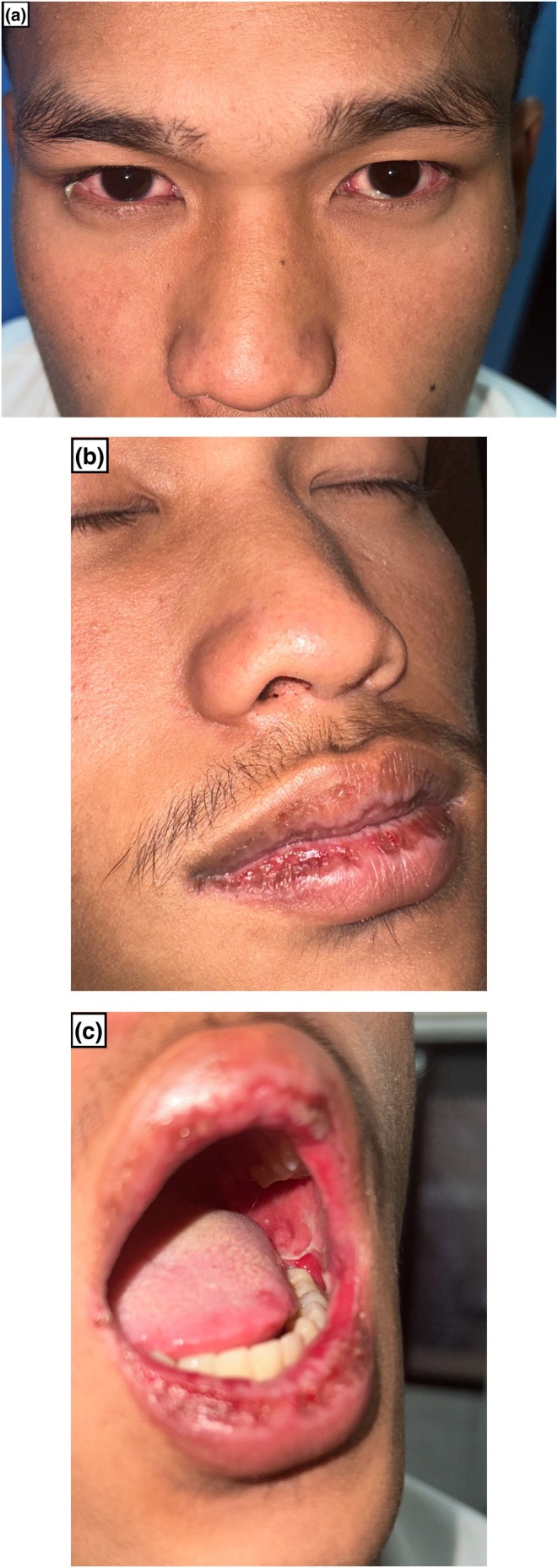
Bilateral conjunctival congestion (a), nasal crusting along with multiple erosions and ulcers over the upper and lower lip and buccal mucosa (b, c).

A 25‐year‐old male presented with intermittent fever and non‐productive cough for 7 days. He developed bilateral conjunctival congestion after 4 days of the onset of fever, for which he was prescribed ocular antibiotics and eye lubricants by an ophthalmologist. The following day, he complained of epistaxis and painful oral lesions and was referred by an otolaryngologist to the dermatology department. There is no history of any medication intake prior to the onset of lesions. He denies simple illness in the past. Mucosae examination revealed conjunctival hyperemia with purulent discharge, nasal crusting, and multiple oral erosions and ulcers over the oral cavity (Figure 1a–c). There were no cutaneous or genital lesions or significant lymphadenopathy, and systemic examination findings were unremarkable.

## 3 Methods

Routine blood investigations revealed leucocytosis with neutrophilia and high C‐reactive protein. The chest X‐ray was normal. On the Tzanck smear, any acantholytic cells or multinucleate giant cells were not seen. Serum IgM for *M. pneumoniae* was positive; however, serology for herpes simplex virus (type 1 and 2) was negative. PCR testing for *M. pneumoniae* was not done as it was not available at our centre.

## 4 Results

**FIGURE 2:**
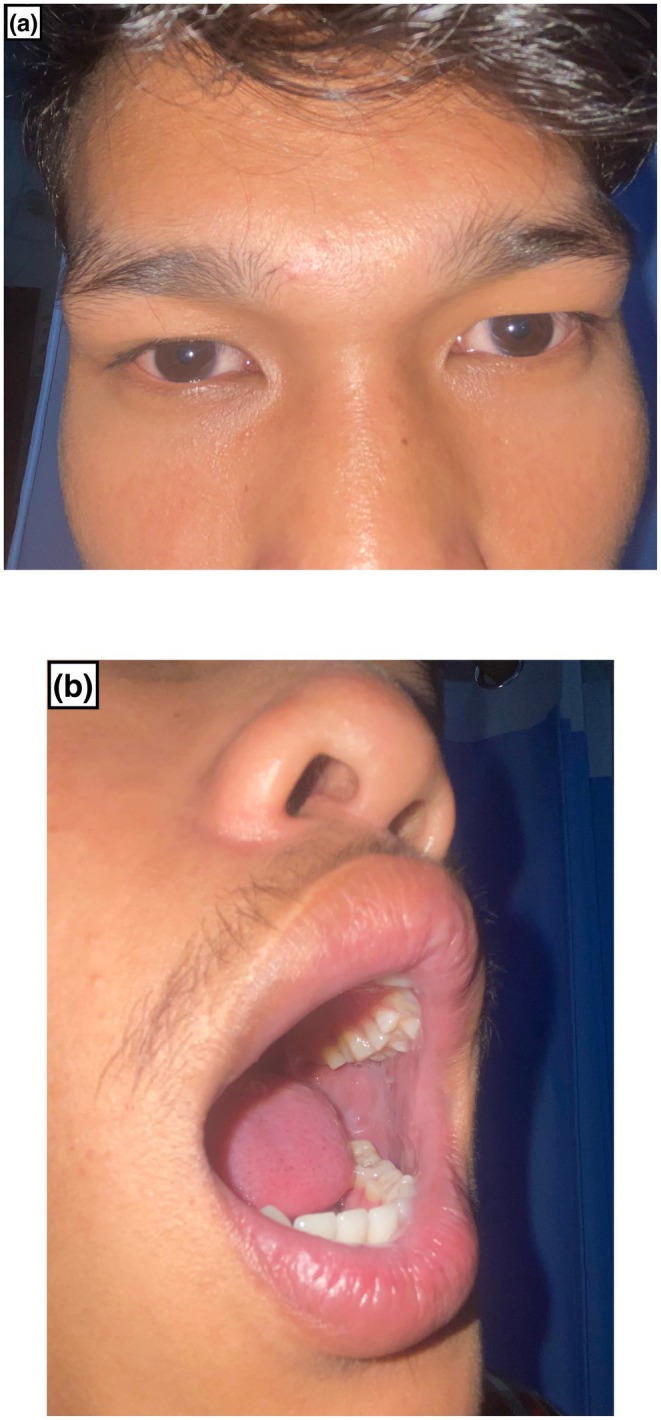
Significant improvement of conjunctival congestion (a), nasal and oral lesions in follow‐up after 10 days (b).

Based on history, clinical examination, and investigations, the diagnosis of *Mycoplasma pneumoniae* ‐associated mucositis was made, and he was prescribed oral azithromycin 500 mg once daily for 5 days along with supportive management. The patient reported significant improvement upon completing the antibiotic course and showed notable progress at the 10‐day follow‐up (Figure 2a,b).

## 5 Discussion

*M*. *pneumoniae* is a common respiratory pathogen that can cause mild upper respiratory tract infections to severe atypical pneumonia, along with non‐pulmonary manifestations like haemolytic anemia, polyarthritis, cutaneous, neurological, cardiac, and hepatic diseases [3]. Direct (presence of *M. pneumoniae* antigen at the site triggering inflammation), indirect (remote *M. pneumoniae* antigen causing immune modulation via immune complex deposition or autoimmunity), and vascular occlusion are the proposed mechanisms for non‐pulmonary manifestations seen with *M. pneumoniae* infections [4]. Central nervous system manifestations are the most frequent extra‐pulmonary complications that present as encephalitis, meningoencephalitis, polyradiculitis, or aseptic meningitis [3]. Cutaneous involvement is seen in 10%–25% of patients and includes non‐specific rashes, erythema multiforme, urticaria, erythema nodosum, Stevens‐Johnson syndrome/toxic epidermal necrolysis, MIRM (*Mycoplasma‐*induced rash and mucositis), MPAM ( *Mycoplasma pneumoniae* ‐associated mucositis), RIME (Reactive infectious mucocutaneous eruption), RIRM (Respiratory infection‐induced rash and mucositis), pityriasis rosea, Reiter syndrome, leukocytoclastic vasculitis, IgA vasculitis, urticarial vasculitis, Raynaud's disease, thrombotic thrombocytopenic purpura, subcorneal pustular dermatosis, Kawasaki disease, Sweet syndrome, and Gianotti‐Crosti syndrome [4, 5, 6].

MIRM (*Mycoplasma‐*induced rash and mucositis) is a relatively new concept and currently, the proposed diagnostic criteria include rash with < 10% detachment of body surface area, 2 or more mucosal sites involved, blisters or flat atypical target lesions, clinical features suggestive of pneumonia, and laboratory values that confirm infection with *M. pneumoniae* (PCR, nasal or oral cultures, and cold agglutinins) [7]. Reactive infectious mucocutaneous eruption (RIME) was recently proposed to replace the term MIRM to account for the fact that non‐ *M. pneumoniae* pathogens like *Chlamydia pneumoniae* , human metapneumovirus, human parainfluenza virus 2, rhinovirus, enterovirus, SARS‐CoV‐2, and influenza B virus may also cause rash and mucositis [8].

MPAM ( *Mycoplasma pneumoniae* ‐associated mucositis), or Fuchs syndrome, a rare variant of SJS characterized by two or more mucosal involvements without any cutaneous lesions, was first described in Germany as “herpes oris conjunctivae.” [2, 9] The pathophysiology is poorly understood; however, it is thought to be due to molecular mimicry between the M pneumoniae P1 adhesin protein and keratinocyte antigens, leading to cross‐reacting autoantibodies against mucosal antigens; immune complex deposition in mucosa and skin activating the complement system or phagocytic cells; or polyclonal activation of B cells and plasma cells to sites of infection leading to altered responses to other unrelated infections [4, 6]. The recurrence tendency and case reports of multiple incidents within families also suggest a possible genetic component [7].

Oral lesions are most frequently seen, ocular lesions in two‐thirds, and genital lesions in three‐fourths of the cases, while nasal mucosa is rarely affected, as seen in our case [1]. Oral lesions are mostly erosions, ulcers, vesicobullae, denudation, and hemorrhagic crusts, while erosions, ulceration, and vesicobullae are seen in urogenital involvement. Ocular involvement may present as conjunctival injection, conjunctivitis, photophobia, eyelid edema, lid margin ulceration, conjunctival pseudomembranes, and, rarely, corneal involvement [1, 4]. MPAM has been reported in children and very rarely in adults, as observed in our case [1].

MPAM is diagnosed clinically and supported by serological and PCR testing [6]. The detection of *M. pneumoniae* from oropharyngeal or polymerase chain reaction (PCR) is a valuable tool for the early and accurate diagnosis, but it is not readily available in developing countries like Nepal [10]. Serological tests can assist in diagnosis, with IgM usually produced within a week of initial infection, peaking between 3 and 6 weeks, then declining. In contrast, a direct IgG response is more common and indicative of reinfection in adult patients [11]. *Differential diagnosis includes HSV‐associated Fuchs syndrome*, *drug‐induced SJS*, *and pemphigus*, *which were excluded by history*, *clinical examination*, *and investigations* [1, 4]. Long‐term sequelae in the mucosal sites are rare, but synechiae and pigmentary changes may occur [2]. A comprehensive approach to management includes supportive, antimicrobial, and immunomodulatory therapies. Treatment with antibiotics such as macrolides, tetracycline, or fluoroquinolones has been shown to limit the pulmonary disease, but it is unclear whether the incidence or severity of the mucocutaneous eruption is reduced. However, we support that early initiation of antibiotics decreases the severity of the mucocutaneous eruption as seen in our case [1, 6]. Our findings align with the systematic review conducted by Vujic et al. which advocates the use of antibiotic therapy in managing MAPM. Their review emphasizes targeting *M. pneumoniae* to eliminate the causative agent, so as to limit the disease severity and duration [11]. An aggressive approach with systemic steroids, cyclosporine, intravenous immunoglobulin, or TNF‐α inhibitors like infliximab and etanercept has also been tried to stop the disease progression and prevent inadvertent complications [2, 4, 9]. Similar to our case, Chorpa et al. reported a case of a 12‐year‐old male with *Mycoplasma pneumoniae* ‐associated Fuchs syndrome affecting oral, ocular, nasal, and genital mucosa. Treatment with azithromycin 500 mg/day and cyclosporine 3 mg/kg/day led to lesion resolution within 10 days [9]. Supportive care, including ocular and mucous membrane care, fluids and nutritional support, and pain control, are necessary [1, 4]. Patients with ocular involvement should be closely monitored by an ophthalmologist. Early treatment with a combination of topical corticosteroid and antibiotic eye drops for conjunctivitis can be beneficial, but amniotic membrane transplantation may be required in some cases [4].

In the absence of cutaneous manifestations, the patient often seeks multiple specialists, including ophthalmologists, dentists, otolaryngologists, gynecologists, or urologists. The differential diagnosis of Fuchs syndrome is broad, so a suspicion of *Mycoplasma* in such cases can avoid delay in diagnosis and adverse sequelae.

## Author Contributions

**Mahesh Mathur:** conceptualization, formal analysis, resources, supervision, validation, visualization, writing – original draft. **Sumit Paudel:** conceptualization, formal analysis, resources, supervision, validation, visualization. **Sambidha Karki:** formal analysis, resources, supervision, visualization, writing – original draft, writing – review and editing. **Nabita Bhattarai:** data curation, investigation, visualization, writing – review and editing. **Shilpa Maharjan:** data curation, investigation, visualization, writing – review and editing. **Sandhya Regmi:** conceptualization, formal analysis, resources, supervision, validation, visualization, writing – original draft.

## Ethics Statement

Reviewed and approved by the Institutional Review Board, College of Medical Sciences (IRBCOMS).

## Consent

The patient in this manuscript has given written informed consent to the publication of his case details.

## Conflicts of Interest

The authors declare no conflicts of interest.

## Data Availability

The data that support the findings of this study are available from the corresponding author upon reasonable request.
